# Open pediatric ureterocelectomy with associated calculus excision and ureteroneocystostomy

**DOI:** 10.1590/S1677-5538.IBJU.2019.0301

**Published:** 2020-09-02

**Authors:** Kevin J. Hebert, Candace Granberg, Patricio Gargollo

**Affiliations:** 1 Department of Urology Mayo Clinic RochesterMN United States Department of Urology, Mayo Clinic, Rochester, MN, United States

## Abstract

**Introduction:**

Pediatric ureteroceles have a cited incidence at autopsy between 1 in 4000 and 1 in 500. In some cases, a calculus may be associated with the ureterocele. This case presentation and surgical video highlights successful management of an intravesical ureterocele and calculus using an open approach.

**Patient and Method:**

A 12 year old male presented to our clinic with irritative LUTS, incomplete emptying, and intermittent incontinence. A urinalysis was positive for blood and follow-up renal ultrasound revealed a 3cm ureterocele with a 1.5cm diameter calculus. Using an open, transabdominal approach, the ureterocele was incised and the calculus was removed. The ureterocele sac was excised at its junction with the bladder, and the distal ureter was released. In a Cohen cross-trigonal approach, a ureteroneocystostomy was then completed.

**Results:**

Our patient tolerated surgery well, and was discharged on post-operative day one. Bladder ultrasound at three months revealed resolution of the ureterocele.

**Conclusion:**

With a post-operative course comparable to robotic surgery, open ureterocelectomy is an important approach that may be utilized in management of complex ureteroceles.

